# Pushing or Pulling Your “Poison”: Clinical Correlates of Alcohol Approach and Avoidance Bias Among Inpatients Undergoing Alcohol Withdrawal Treatment

**DOI:** 10.3389/fpsyg.2021.663087

**Published:** 2021-05-25

**Authors:** Hugh Piercy, Victoria Manning, Petra K. Staiger

**Affiliations:** ^1^Monash Addiction Research Centre, Eastern Health Clinical School, Monash University, Melbourne, VIC, Australia; ^2^Turning Point, Eastern Health, Melbourne, VIC, Australia; ^3^School of Psychology, Deakin University, Geelong, VIC, Australia; ^4^Centre for Drug Use, Addictive and Antisocial Behaviour Research, Deakin University, Geelong, VIC, Australia

**Keywords:** approach bias, avoidance bias, cognitive bias modification, approach bias modification, alcohol use disorder, measurement

## Abstract

**Introduction:** Alcohol approach bias, the tendency to automatically move toward alcohol cues, has been observed in people who drink heavily. However, surprisingly, some alcohol-dependent patients demonstrate an alcohol avoidance bias. This inconsistency could be explained by the clinical or demographic profile of the population studied, yet this has not been examined in approach bias modification (ABM) trials to date. We aimed to determine the proportion of patients with an approach or avoidance bias, assess whether they differ on demographic and drinking measures, and to examine the clinical correlates of approach bias.

**Method:** These research questions were addressed using baseline data from 268 alcohol-dependent patients undergoing inpatient withdrawal treatment who then went on to participate in a trial of ABM.

**Results:** At trial entry (day 3 or 4 of inpatient withdrawal), 155 (57.8%) had an alcohol approach bias and 113 (42.2%) had an avoidance bias. These two groups did not differ on any demographic or relevant drinking measures. Approach bias was significantly and moderately associated with total standard drinks consumed in the past 30 days (*r* = 0.277, *p* = 0.001) but no other indices of alcohol consumption or problem severity.

**Conclusion:** Whilst the majority of alcohol-dependent patients showed an alcohol approach bias, those with an avoidance bias did not differ in demographic or clinical characteristics, and the strength of approach bias related only to recent consumption. Further research is needed to develop more accurate and personally tailored measures of approach bias, as these findings likely reflect the poor reliability of standard approach bias measures.

## Introduction

Contemporary neurocognitive models of addiction posit that automatic cognitive processes play a critical role in the maintenance of alcohol use disorders (AUDs) (Stacy and Wiers, 2010). Research has demonstrated that alcohol-related cues can automatically capture attention (i.e., known as an “attentional bias”; Field and Cox, 2008), and that these cues can trigger automatic action tendencies to approach alcohol (i.e., known as an “approach bias”; Field et al., 2008). Alcohol-related attentional and approach biases are thought to develop through extended periods of frequent drinking, which involve numerous associative learning experiences in which the rewarding effects of alcohol are paired with various alcohol-related cues (Field and Cox, 2008). These associations become sensitised (i.e., very easily, rapidly, and strongly activated) such that re-exposure to these cues (or even memory of them) is quickly and easily able to activate mental representations of alcohol's desired effects, influencing attentional and approach biases (Stacy and Wiers, 2010) and leading to alcohol consumption (Field and Cox, 2008; Martin-Braunstein et al., 2016). Whilst alcohol-dependent individuals are theorised to demonstrate an approach bias for alcohol-related cues (Field et al., 2008), there is inconsistent evidence about the proportion who actually do (Spruyt et al., 2013; Ernst et al., 2014), and studies have failed to investigate whether the presence of approach bias is limited to individuals with certain clinical or demographic profiles.

Approach bias is typically measured through behavioural reaction tasks which assess whether individuals are faster to respond to substance-related stimuli displayed on a computer screen compared to their response to neutral stimuli. Commonly used tasks are the approach avoidance task (AAT; Rinck and Becker, 2007), in which participants move stimuli towards (approach) and away (avoid) from themselves using a joystick, and the stimulus–response compatibility task (SRC; De Houwer et al., 2001), where participants perform a symbolic movement by making a manikin walk towards (approach) or away (avoid) from stimuli (Kersbergen et al., 2015). Faster RTs for approaching vs. avoiding alcohol stimuli relative to non-alcohol stimuli indicate an approach bias (positive score), and the opposite indicates an avoidance bias (negative score; Kersbergen et al., 2015). Both relevant-feature (R) and irrelevant-feature (IR) versions of the AAT and SRC exist, where relevant-feature tasks instruct participants to respond to the explicit contents of the presented stimuli (e.g., avoid images containing alcohol, approach images containing soft-drinks), whilst irrelevant-feature tasks provide a more implicit assessment of approach bias by requiring participants to respond to an extraneous image feature (e.g., avoid images presented in landscape orientation, approach images presented in portrait orientation; De Houwer, 2003; Wiers et al., 2009, 2017).

People experiencing alcohol problems have been shown to demonstrate stronger alcohol approach bias compared to those without problems (Sharbanee et al., 2013; Ernst et al., 2014; Wiers et al., 2014). For example, Ernst et al. (2014) compared 21 alcohol-dependent inpatients to 21 matched controls and found that patients demonstrated a stronger alcohol approach bias compared to controls, and that patients demonstrated stronger neural activations of reward circuitry when approaching rather than avoiding alcohol pictures, where the reverse was found for healthy controls (Ernst et al., 2014). Alcohol approach bias has also been associated with higher levels of self-reported craving and weekly alcohol consumption (Field et al., 2008). However, contrary to predictions, some studies have found that alcohol-dependent patients in residential settings report an overall avoidance bias at baseline (Spruyt et al., 2013; Snelleman et al., 2015; Field et al., 2017; Rinck et al., 2018). For example, one study with 40 abstaining alcohol-dependent patients (18–21 days after drinking) found that they demonstrated a relative alcohol avoidance bias at baseline (using the R-SRC; Spruyt et al., 2013), while another study found that participants (120 recently detoxified alcohol-dependent patients) demonstrated no overall mean approach or avoidance bias at baseline (Field et al., 2017). However, it is worth noting that performance on the R-SRC is not correlated with performance on the IR-AAT (Wiers et al., 2013), which could explain the differences in findings.

We recently conducted a large trial of approach bias modification (ABM) where we trained alcohol-dependent patients to push away alcohol cues and hence reduce their alcohol approach bias (Manning et al., 2021). We found that these participants had a mean approach bias on the IR-AAT at baseline, which is consistent with some other large ABM trials of alcohol-dependent patients (Wiers et al., 2011; Eberl et al., 2013). In none of these studies do we know what proportion of participants had an avoidance or approach bias on treatment entry (since only mean approach bias scores are reported), nor do we know whether those with an approach or avoidance bias exhibited any clinical or demographic differences. This would be useful for identifying suitable targets for ABM. Given the growing adoption of ABM into standard alcohol treatment practise guidelines (Mann et al., 2017; Haber, 2021), addressing these knowledge gaps is warranted.

This study aimed to determine the proportion of alcohol-dependent patients with an alcohol approach bias and an alcohol avoidance bias (using the IR-AAT) in a large inpatient sample and to examine whether these two groups exhibit any demographic or clinical differences in the early stages of treatment. Additionally, we aimed to examine whether approach/avoidance bias scores on the IR-AAT were associated with any clinical indices of AUD problem severity. Specifically, we hypothesised that a greater proportion of participants would demonstrate an alcohol approach bias at baseline, and that these participants would demonstrate greater indices of alcohol use (i.e., drinking days, heavy drinking days and standard drinks in past month) and problem severity (i.e., number of previous withdrawal treatment episodes, duration of problematic alcohol use, craving, severity of dependence) compared to those with an avoidance bias. Additionally, we hypothesised that the strength of approach bias (i.e., higher scores on the IR-AAT) would be significantly correlated with indices of use and problem severity among those with a baseline approach bias.

## Method

### Participants

The full sample from the existing RCT, conducted in alcohol residential withdrawal units (Manning et al., 2021), consisted of 300 participants. However, for the purposes of these analyses, we included only the 268 participants who completed a baseline assessment of alcohol approach bias. Participants were recruited from four inpatient withdrawal treatment units in Melbourne, Australia between 2017 and 2019. Participants were required to be aged between 18 and 65; to meet Diagnostic and Statistical Manual of Mental Disorders, Fifth Edition (DSM-5) criteria for moderate or severe AUD (American Psychiatric Association, 2013); to have used alcohol at least weekly in the month prior to admission to inpatient withdrawal treatment; and to be planning to stay in treatment long enough to complete the 4-day ABM (treatment) or Sham (control) training protocol of the larger RCT (Manning et al., 2021). Patients were excluded if they had a diagnosed history of neurological illness or injury, concussion resulting in loss of consciousness longer than 30 min or any diagnosed intellectual disability, or if they were assessed by clinical staff to be too acutely unwell to provide informed consent or participate. Participants provided written informed consent. Summary statistics are provided in Table 1.

**Table 1:** Baseline sample characteristics and differences between participants with a baseline alcohol approach bias relative to an alcohol avoidance bias (*N* = 268).

**Variable**	***N*^**a**^**	**Whole sample**	**Alcohol approach bias (*n* = 155)**	**Alcohol avoidance bias (*n* = 113)**	***p*^**b**^**
Age, mean (SD)	268	43.3 (10.4)	43.0 (11.0)	43.8 (9.6)	0.534
Years of problematic alcohol use, mean (SD)^c^	267	17.0 (11.0)	16.6 (11.2)	17.7 (10.8)	0.418
Number of previous withdrawal treatment episodes, mean (SD)	268	2.5 (3.9)	2.6 (4.2)	2.4 (3.5)	0.622
Years of education, mean (SD)	266	12.5 (2.6)	12.5 (2.4)	12.5 (2.8)	0.921
Drinking days in past month, mean (SD)^d^	268	27.2 (5.1)	27.0 (5.3)	27.6 (4.6)	0.310
Heavy drinking days in past month, mean (SD)	259	26.5 (5.9	26.2 (6.3)	27.0 (5.3)	0.308
Standard drinks in past month, mean (SD)	265	575.3 (325.3)	584.4 (338.4)	562.9 (307.5)	0.595
VAS craving score, mean (SD)	268	31.4 (26.6)	31.3 (26.8)	31.7 (26.5)	0.906
ACQ total score, mean (SD)	268	3.9 (1.4)	3.8 (1.4)	4.0 (1.4)	0.209
SADQ total score, mean (SD)	242	32.3 (11.8)	32.7 (11.7)	31.7 (12.0)	0.509
Gender, male %	268	56.3	54.8	58.4	0.605
Born in Australia, %	268	83.6	85.2	81.4	0.414
Aboriginal or Torres Strait Islander, %	268	6.3	5.2	8.0	0.352
Unemployed, %	263	74.5	75.5	73.2	0.674
Unstable housing, %	267	16.5	14.2	19.6	0.236
PDOC was alcohol, %	268	95.5	97.4	92.9	0.176
Family history of SUD, %	264	56.4	56.2	56.8	0.929
Psychiatric disorder, %	268	29.9	80.6	78.8	0.704

a *N values are displayed due to missing data for some variables*.

b *Statistical difference between those with an alcohol approach bias vs. alcohol avoidance bias*.

c *This was calculated as the difference between participants' current age and self-reported age of onset of problematic alcohol use*.

d *Median score was 30, where 58.2% of participants consumed alcohol on 30 of the past 30 days*.

*VAS, Visual Analogue Scale; ACQ, Alcohol Craving Questionnaire; SADQ, Severity of Alcohol Dependence Questionnaire; PDOC, Primary Drug of Concern; SUD, Substance Use Disorder*.

### Measures

#### Clinical and Demographic Questionnaire

Prior to completing the IR-AAT, researchers administered a questionnaire at baseline assessing participants' age, gender, employment status, housing status, country of birth, Aboriginal or Torres Strait Islander status, education history, years of problematic alcohol use, number of previous withdrawal treatment episodes, primary and secondary drugs of concern, family history of substance use disorder, and psychiatric history.

#### Timeline Follow-Back

The timeline follow-back (TLFB; Sobell and Sobell, 1996) was used to measure alcohol consumption and captured drinking days, heavy drinking days (defined as consuming ≥5 standard drinks for females or ≥6 standard drinks for males) and total standard drinks at baseline (covering the 30 days preceding inpatient admission). The TLFB is a widely used interview method for estimating alcohol use and has been shown to concur well with other measures of alcohol use in previous research (Simons et al., 2015).

#### Alcohol Craving Questionnaire—Short Form—Revised

The Alcohol Craving Questionnaire—Short Form—Revised (ACQ-SF-R) was used to assess current cravings for alcohol. The scale contains 12 items from the 47-item Alcohol Craving Questionnaire (ACQ-NOW; Singleton et al., 1995), which are strongly correlated with the ACQ-NOW and its four subscales (compulsivity, expectancy, purposefulness, and emotionality). Scores are summed to give a total score or can be summed to yield individual subscale scores, where higher scores indicate stronger cravings (Tiffany et al., 2000).

#### Severity of Alcohol Dependence Questionnaire

The Severity of Alcohol Dependence Questionnaire (SADQ; Stockwell et al., 1994) is a 20-item questionnaire which assesses symptoms of alcohol dependence, including physical withdrawal, affective withdrawal and drinking to relieve withdrawal symptoms. Higher scores indicate greater severity of alcohol dependence, and the SADQ has been shown to demonstrate good concurrent validity and test–retest reliability (Stockwell et al., 1983).

#### Irrelevant-Feature Alcohol Approach Avoidance Task

An assessment version of the IR-AAT was used to assess alcohol approach bias (Wiers et al., 2009). Using a laptop and a joystick, participants were presented with a series of images in landscape or portrait orientation (10 alcohol-related images and 10 non-alcohol-related images, each repeated 2 times, yielding a total of 40 image presentations) and instructed to push away (avoid) landscape images and pull (approach) portrait images. Each image type (alcoholic or non-alcoholic) appeared in landscape and portrait orientation 50% of the time. Pushing and pulling the joystick caused the images to decrease and increase in size, respectively. Incorrect responses were followed by a red “X,” and participants were required to correct their response in order to proceed with the task.

Trials were considered valid if the initial joystick response was correct and the reaction time was between 300 and 3,000 ms. If at least 70% of the trials were valid (i.e., at least seven of the 10 trials for any picture-response category were correct), median reaction times were calculated separately for each of the four picture-response categories (alcohol-pull, alcohol-push, non-alcohol-pull, non-alcohol-push). If <70% of the trials were valid, the median for that picture-response category was considered missing. Approach bias was calculated separately for alcohol-related trials and non-alcohol-related trials by subtracting the median reaction time for pull responses from the median reaction time for push responses. The non-alcohol approach bias scores were then subtracted from the alcohol approach bias scores to provide an index of alcohol approach bias relative to non-alcohol-related images. Internal consistency of the IR-AAT was calculated using the method reported by Kersbergen et al. (2015). The internal consistency was low (Cronbach's α = 0.35 for alcohol-related items and 0.34 for non-alcohol-related items), and the test–retest reliability (calculated only for the 117 participants in the sham training control condition who completed both baseline and post-test AAT assessments in the larger RCT) was poor (*r* = 0.027, *p* = 0.774; see Manning et al., 2021).

### Procedure

Participants were screened on admission for eligibility by clinicians at the participating withdrawal treatment sites and referred to a member of the research team if interested in participating. Participants provided consent and completed baseline assessments on day 3 of their 7-day inpatient admission (*M* = 7.3 days, *SD* = 2.6). The baseline assessments included a baseline questionnaire which assessed eligibility, demographic and clinical characteristics, TLFB, SADQ, ACQ, and the AAT. The first session of ABM was typically conducted either on the same day as the baseline questionnaires or the following day. This study was approved by the St. Vincent's Hospital Melbourne Human Research Ethics Committee (HREC; reference number 030/17) and the Monash University HREC (project number 8447).

## Results

Of the 268 participants who completed the baseline AAT, 155 (57.8%) had an alcohol approach bias compared to 113 (42.2%) who had an avoidance bias. Overall, the sample had an approach bias for alcohol cues relative to non-alcohol cues (*M* = 34.56, 95% bootstrapped CI = 5.71–62.22; *SD* = 243.77). There were no significant differences between those with an alcohol approach bias compared to those with an avoidance bias on any of the clinical or demographic variables analysed (see Table 1).

Pearson's bivariate correlations were used to analyse whether alcohol approach bias was associated with indices of AUD severity among participants with an alcohol approach bias. See Table 2 for the correlation matrix. There was a significant, moderate association between alcohol approach bias and the number of standard drinks consumed in the past 30 days (*r* = 0.277, *p* = 0.001, *n* = 153); however, associations with all other indices of AUD severity did not reach significance. Among those participants who possessed an avoidance bias at baseline (*n* = 113), the association between bias score and standard drinks was non-significant (*r* = 0.033, *p* = 0.729), and there were no associations with other indices of AUD severity that approached significance. Finally, the relationship between standard drinks and relative alcohol approach bias among those with a baseline approach bias was further confirmed in a multiple regression analysis, where standard drinks emerged as the only significant predictor of approach bias (*B* = 0.182, *t* = 3.05, *p* = 0.003), model summary: *F*_(7, 124)_ = 1.99, *p* = 0.062, *R*^2^ = 0.05, *f* ^2^ = 0.112.

**Table 2:** Pearson correlation matrix for indices of alcohol consumption and problem severity among those with an approach bias (*N* = 155).

	**1**	**2**	**3^**a**^**	**^**a**^**	**5^**a**^**	**6**	**7**	**8**
1. Alcohol approach bias	-							
2. Standard drinks in past month	0.277^**^	-						
3. Drinking days^a^	0.048	0.377^**^	-					
4. Heavy drinking days^a^	0.107	0.524^**^	0.877^**^	-				
5. Number of previous withdrawal treatment episodes^a^	−0.096	0.124	−0.128	−0.045	-			
6. Years of problematic alcohol use	0.073	0.137	0.010	0.065	0.129^*^	-		
7. SADQ total score	0.015	0.382^**^	0.060	0.191^**^	0.299^**^	0.080	-	
8. ACQ total score	0.120	0.117	0.023	0.024	0.089	−0.094	0.187^*^	-

a *Spearman's rho is displayed due to skewed data; SADQ, Severity of Alcohol Dependence Questionnaire; ACQ, Alcohol Craving Questionnaire*.

** *Correlation is significant at the 0.01 level*.

* *Correlation is significant at the 0.05 level*.

## Discussion

The present study sought to examine the presence of alcohol approach biases and their clinical correlates in a large sample of alcohol-dependent inpatients undergoing withdrawal treatment. In support of our first hypothesis, we found that just over half of the participants (57.8%) had an IR-AAT score indicative of an alcohol approach bias, and that the overall sample mean was also indicative of an alcohol approach bias. However, participants with an approach bias did not significantly differ on any of the demographic or clinical variables analysed from those with an avoidance bias. In contrast to our prediction for the second hypothesis, we found that the alcohol approach bias score was significantly associated with only past-month alcohol consumption and no other indices of consumption or problem severity among those with a baseline approach bias.

The finding that majority of participants and the overall sample mean demonstrated an approach bias supports a number of patient studies (Wiers et al., 2011, 2014; Eberl et al., 2013; Ernst et al., 2014) but contrasts with those reporting a mean avoidance bias (Spruyt et al., 2013) or an absence of an approach or avoidance bias altogether (Field et al., 2017). This inconsistency could be explained by the different approach bias measures used in these studies, where Field et al. (2017) and Spruyt et al. (2013) used the R-SRC rather than the IR-AAT (Spruyt et al., 2013; Field et al., 2017). As noted by Wiers et al. (2013), it is possible that the type of avoidance associations measured by the R-SRC is distinct from those captured by the IR-AAT and may be more strongly related to predicting relapse rather than those which are assessed and retrained using the IR-AAT (Wiers et al., 2013).

The absence of any group differences in demographic or clinical variables was unexpected, as was the finding that past-month alcohol consumption and no other indices of consumption or problem severity were correlated with the strength of approach bias. Whilst this supports early work by Field et al. (2008), where approach bias (on the R-SRC) was associated with weekly alcohol consumption, we failed to replicate their finding that approach bias is associated with alcohol cravings (Field et al., 2008). This was surprising, given the theoretical mechanism through which approach bias arises (i.e., through repeated associative learning experiences), whereby those who drink more frequently, in larger quantities, with longer durations of problem drinking and more extensive treatment involvement would be expected to demonstrate stronger approach bias. This may suggest that the presence of approach bias is state-dependent or varies depending on recent alcohol consumption (even among individuals with severe AUD), and future research would benefit from investigating how approach bias fluctuates over time and what factors influence this process.

These inconsistencies could also be due to the notoriously poor reliability of approach bias measures themselves (Kersbergen et al., 2015; Rinck et al., 2018), where we also found poor internal consistency and test–retest reliability in the version of the IR-AAT used in the present study. This measurement error may be at least partly due to the task comprising only half the number of trials used in standard IR-AAT tasks (in order to minimise excessive participant exposure to alcohol images during the vulnerable early withdrawal phase). The poor internal consistency could also be because individuals might only demonstrate an approach bias to alcohol cues that reflect the drinks they regularly consume. Since approach bias is posited to arise through repeated associative learning experiences, it is possible that individuals will only show an approach bias to certain alcoholic beverages that they mostly commonly consume (or at least show a much stronger bias towards these beverages). In our research, we have found that participants typically consume only a narrow range of alcoholic beverages, and given that measures of approach bias use a standard set of alcohol-related images, only a minority of the presented images may elicit responses that are indicative of an approach bias. For example, a participant who only consumes wine may demonstrate a stronger approach bias towards wine-related cues compared to other alcohol-related cues for beverages that they do not drink (e.g., beer or spirits). Indeed, studies have shown that craving and associated psychophysiological indices are stronger in response to alcohol cues that strongly resemble the most commonly consumed beverage (Staiger and White, 1991).

Researchers have begun trialling personalisation in ABM (Manning et al., 2020; Garfield et al., 2021) where participants select or rate alcohol/drug cues that best represent the substances they frequently consume, which subsequently comprise the avoidance stimuli in the training task. Personalising approach bias assessment may therefore provide a more accurate assessment of an individual's alcohol approach bias. Future research may also profit from exploring new ways of measuring approach bias (e.g., virtual reality; Eiler et al., 2019) and should explore how conventional measures of approach bias correlate with objective biomarkers of psychophysiological arousal.

Our findings should be considered in light of the study limitations. Firstly, as previously mentioned, our measure of approach bias contained only half the recommended number of trials and demonstrated poor reliability. Secondly, it is important to note that baseline approach bias was typically assessed on day 3 of inpatient withdrawal treatment when participants were receiving much higher doses of benzodiazepines than at post-training assessment, affecting reaction times, which could explain the poor test–retest reliability observed. A third limitation is that alcohol approach bias was calculated relative to non-alcohol approach bias (thereby indicating the strength of an individual's bias towards alcohol beverage cues compared to non-alcohol beverage cues). It was assumed that the non-alcohol beverage cues represent truly neutral stimuli; however, it is possible that some of the beverage types and brands may still have elicited appetitive responses, particularly when these soft drinks are commonly associated with alcoholic beverages (e.g., bottles of Coke for a whiskey and Coke drinker).

Despite these limitations, this paper addresses important questions regarding the measurement of approach bias in alcohol-dependent patients and identifies key considerations for future research. Whilst it may be desirable to administer ABM only to those patients with an alcohol approach bias, the absence of mediation effects in most clinical trials to date (Wiers et al., 2011; Rinck et al., 2018; Manning et al., 2021), coupled with its clear effects on relapse reduction (Wiers et al., 2011; Eberl et al., 2013; Rinck et al., 2018; Manning et al., 2021), suggests that ABM should be routinely offered to all alcohol-dependent patients—particularly given its low-cost and short administration time. Most importantly, this paper raises some important questions regarding the measurement and clinical relevance of approach bias and highlights the need to develop more accurate measures (e.g., through personally tailored measures or virtual reality paradigms).

## Data Availability Statement

The datasets presented in this article are not readily available because longer term outcomes of the broader trial have yet to be completed, hence the full dataset is currently unavailable. Requests to access the datasets should be directed to Victoria.manning@monash.edu.

## Ethics Statement

The studies involving human participants were reviewed and approved by the St. Vincent's Hospital Melbourne Human Research Ethics Committee (reference number 030/17) and the Monash University HREC (project number 8447). The patients/participants provided their written informed consent to participate in this study.

## Author Contributions

HP wrote the first draft with VM and conducted the analyses. PS provided input and overall guidance regarding the structure of the paper. All authors conceptualised the current paper.

## Conflict of Interest

The authors declare that the research was conducted in the absence of any commercial or financial relationships that could be construed as a potential conflict of interest.

## References

[B1] American Psychiatric Association (2013). Diagnostic and Statistical Manual of Mental Health Disorders, 5th Edn. Arlington, VA: American Psychiatric Association.

[B2] De Houwer J. (2003). A structural analysis of indirect measures of attitudes, in The Psychology of Evaluation, eds Musch J. Klauer K. (Mahwah, NJ: Lawrence Erlbaum Associates Publishers), 219–244.

[B3] De Houwer J. Crombez G. Baeyens F. Hermans D. (2001). On the generality of the affective Simon effect. Cogn. Emot. 15, 189–206. 10.1080/02699930125883

[B4] Eberl C. Wiers R. W. Pawelczack S. Rinck M. Becker E. S. Lindenmeyer J. (2013). Approach bias modification in alcohol dependence: do clinical effects replicate and for whom does it work best? Dev. Cogn. Neurosci. 4, 38–51. 10.1016/j.dcn.2012.11.002 23218805 PMC6987692

[B5] Eiler T. J. Grunewald A. Machulska A. Klucken T. Jahn K. Niehaves B. (2019). A preliminary evaluation of transferring the approach avoidance task into virtual reality, in Paper Presented at the International Conference on Information Technologies in Biomedicine (Kamień Slaski).

[B6] Ernst L. H. Plichta M. M. Dresler T. Zesewitz A. K. Tupak S. V. Haeussinger F. B.. (2014). Prefrontal correlates of approach preferences for alcohol stimuli in alcohol dependence. Addict. Biol. 19, 497–508. 10.1111/adb.12005 23145772

[B7] Field M. Cox W. M. (2008). Attentional bias in addictive behaviors: a review of its development, causes, and consequences. Drug Alcohol Depend. 97, 1–20. 10.1016/j.drugalcdep.2008.03.030 18479844

[B8] Field M. Di Lemma L. Christiansen P. Dickson J. (2017). Automatic avoidance tendencies for alcohol cues predict drinking after detoxification treatment in alcohol dependence. Psychol. Addict. Behav. 31, 171–179. 10.1037/adb0000232 27935726 PMC5343749

[B9] Field M. Kiernan A. Eastwood B. Child R. (2008). Rapid approach responses to alcohol cues in heavy drinkers. J. Behav. Ther. Exp. Psychiatry 39, 209–218. 10.1016/j.jbtep.2007.06.001 17640615

[B10] Garfield J. B. B. Piercy H. Arunogiri S. Lubman D. I. Campbell S. C. Sanfilippo P. G.. (2021). Protocol for the methamphetamine approach-avoidance training (MAAT) trial, a randomised controlled trial of personalised approach bias modification for methamphetamine use disorder. Trials 22:21. 10.1186/s13063-020-04927-6 33407781 PMC7788914

[B11] Haber P. (2021). Relapse prevention, aftercare, and long-term follow-up, in Australian Guidelines for the Treatment of Alcohol Problems, eds Haber P. Riordan B. C. (Canberra, ACT: Commonwealth Department of Health).

[B12] Kersbergen I. Woud M. L. Field M. (2015). The validity of different measures of automatic alcohol action tendencies. Psychol. Addict. Behav. 29, 225–230. 10.1037/adb0000009 25134039

[B13] Mann K. Batra A. Fauth-Buhler M. Hoch E. The Guideline Group (2017). German guidelines on screening, diagnosis, and treatment of alcohol use disorders. Eur. Addict. Res. 23, 45–60. 10.1159/000455841 28178695

[B14] Manning V. Garfield J. B. B. Staiger P. K. Lubman D. I. Lum J. A. G. Reynolds J.. (2021). Effect of cognitive bias modification on early relapse among adults undergoing inpatient alcohol withdrawal treatment: a randomized clinical trial. JAMA Psychiatry 78, 133–140. 10.1001/jamapsychiatry.2020.3446 33146693 PMC7643044

[B15] Manning V. Piercy H. Garfield J. B. B. Lubman D. I. (2020). Personalized approach bias modification smartphone app (“SWIPE”) to reduce alcohol use among people drinking at hazardous or harmful levels: protocol for an open-label feasibility study. JMIR Res. Protoc. 9:e21278. 10.2196/21278 32795989 PMC7455870

[B16] Martin-Braunstein L. Kuerbis A. Ochsner K. Morgenstern J. (2016). Implicit alcohol approach and avoidance tendencies predict future drinking in problem drinkers. Alcohol Clin. Exp. Res. 40, 1945–1952. 10.1111/acer.13151 27421061 PMC6299832

[B17] Rinck M. Becker E. S. (2007). Approach and avoidance in fear of spiders. J. Behav. Ther. Exp. Psychiatry 38, 105–120. 10.1016/j.jbtep.2006.10.001 17126289

[B18] Rinck M. Wiers R. W. Becker E. S. Lindenmeyer J. (2018). Relapse prevention in abstinent alcoholics by cognitive bias modification: clinical effects of combining approach bias modification and attention bias modification. J. Consult. Clin. Psychol. 86, 1005–1016. 10.1037/ccp0000321 30507226

[B19] Sharbanee J. M. Stritzke W. G. Wiers R. W. Young P. Rinck M. MacLeod C. (2013). The interaction of approach-alcohol action tendencies, working memory capacity, and current task goals predicts the inability to regulate drinking behavior. Psychol. Addict. Behav. 27, 649–661. 10.1037/a0029982 23088407

[B20] Simons J. S. Wills T. A. Emery N. N. Marks R. M. (2015). Quantifying alcohol consumption: self-report, transdermal assessment, and prediction of dependence symptoms. Addict. Behav. 50, 205–212. 10.1016/j.addbeh.2015.06.042 26160523 PMC4523087

[B21] Singleton E. G. Tiffany S. T. Henningfield J. E. (1995). Development and validation of a new questionnaire to assess craving for alcohol, in Paper Presented at the Proceedings of the 56th Annual Meeting (Rockville, MD: The College on Problems of Drug Dependence, Inc.).

[B22] Snelleman M. Schoenmakers T. M. van de Mheen D. (2015). Attentional bias and approach/avoidance tendencies do not predict relapse or time to relapse in alcohol dependency. Alcohol Clin. Exp. Res. 39, 1734–1739. 10.1111/acer.12817 26247388

[B23] Sobell L. C. Sobell M. B. (1996). Timeline Followback User's Guide: A Calendar Method for Assessing Alcohol and Drug Use. Toronto, ON: Addiction Research Foundation.

[B24] Spruyt A. De Houwer J. Tibboel H. Verschuere B. Crombez G. Verbanck P.. (2013). On the predictive validity of automatically activated approach/avoidance tendencies in abstaining alcohol-dependent patients. Drug Alcohol Depend. 127, 81–86. 10.1016/j.drugalcdep.2012.06.019 22776440

[B25] Stacy A. W. Wiers R. W. (2010). Implicit cognition and addiction: a tool for explaining paradoxical behavior. Annu. Rev. Clin. Psychol. 6, 551–575. 10.1146/annurev.clinpsy.121208.131444 20192786 PMC3423976

[B26] Staiger P. K. White J. M. (1991). Cue reactivity in alcohol abusers: stimulus specificity and extinction of the responses. Addict. Behav. 16, 211–221. 10.1016/0306-4603(91)90014-9 1776539

[B27] Stockwell T. Murphy D. Hodgson R. (1983). The severity of alcohol dependence questionnaire: its use, reliability and validity. Br. J. Addict. 78, 145–155. 10.1111/j.1360-0443.1983.tb05502.x 6135435

[B28] Stockwell T. Sitharthan T. McGrath D. Lang E. (1994). The measurement of alcohol dependence and impaired control in community samples. Addiction 89, 167–174. 10.1111/j.1360-0443.1994.tb00875.x 8173482

[B29] Tiffany S. T. Carter B. L. Singleton E. G. (2000). Challenges in the manipulation, assessment, and interpretation of craving relevant variables. Addiction 95(Suppl. 2), S177–S187. 10.1046/j.1360-0443.95.8s2.7.x 11002913

[B30] Wiers C. E. Gladwin T. E. Ludwig V. U. Gropper S. Stuke H. Gawron C. K.. (2017). Comparing three cognitive biases for alcohol cues in alcohol dependence. Alcohol Alcohol. 52, 242–248. 10.1093/alcalc/agw063 28182202

[B31] Wiers C. E. Stelzel C. Park S. Q. Gawron C. K. Ludwig V. U. Gutwinski S.. (2014). Neural correlates of alcohol-approach bias in alcohol addiction: the spirit is willing but the flesh is weak for spirits. Neuropsychopharmacology 39, 688–697. 10.1038/npp.2013.252 24060832 PMC3895246

[B32] Wiers R. W. Eberl C. Rinck M. Becker E. S. Lindenmeyer J. (2011). Retraining automatic action tendencies changes alcoholic patients' approach bias for alcohol and improves treatment outcome. Psychol. Sci. 22, 490–497. 10.1177/0956797611400615 21389338

[B33] Wiers R. W. Gladwin T. E. Rinck M. (2013). Should we train alcohol-dependent patients to avoid alcohol? Front. Psychiatry 4:33. 10.3389/fpsyt.2013.00033 23734131 PMC3660696

[B34] Wiers R. W. Rinck M. Dictus M. van den Wildenberg E. (2009). Relatively strong automatic appetitive action-tendencies in male carriers of the OPRM1 G-allele. Genes Brain Behav. 8, 101–106. 10.1111/j.1601-183X.2008.00454.x 19016889

